# Water insecurity potentially undermines dietary diversity of children aged 6–23 months: Evidence from India

**DOI:** 10.1111/mcn.12929

**Published:** 2020-01-30

**Authors:** Neetu Choudhary, Roseanne Schuster, Alexandra Brewis, Amber Wutich

**Affiliations:** ^1^ Center for Global Health, School of Human Evolution and Social Change Arizona State University Tempe Arizona

**Keywords:** child nutrition, dietary diversity, gender, India, water access, water availability

## Abstract

Dietary diversity is a crucial pathway to child nutrition; lack of diversity may deprive children of critical macro and micronutrients. Though water along with hygiene and sanitation is a known driver of child undernutrition, a more direct role of household water in shaping dietary diversity remains unexplored. Existing literature provides a sound theoretical basis to expect that water could affect dietary diversity among young children. Here, we test the proposition that suboptimal household access to water and low regional water availability associate with lower dietary diversity among young children. Using the nationally representative 2015–2016 India Demographic and Health Survey data, we conducted a probit analysis on the sample of 69,841 children aged 6–23 months to predict the probability that a child achieves minimum standards of dietary diversity (MDD). After controlling for relevant socioeconomic and gender‐related covariates, we found that children in household with suboptimal household water access were two percentage points less likely to achieve MDD, when compared with those from households with optimal water access. Children in high water availability regions had nine percentage points greater probability of achieving MDD compared with children from low water availability regions, accounting for household water access. As dietary diversity is central to nutrition, establishing the role of water access in shaping early childhood dietary diversity broadens the framework on how household material poverty shapes child malnutrition—independent of sanitation and hygiene pathways. This provides additional window for nutrition planning and intervention wherein water‐based strategies can be leveraged in multiple ways.

**Key messages**

- Suboptimal household water access is associated with failure to meet minimum dietary diversity standards among children aged 6–23 months in India.
- Children living in regions of higher water availability are relatively buffered.
- This role of water in shaping access to dietary diversity among children can be leveraged for nutritional improvements, given that dietary diversity is central to child nutrition.

## 1 INTRODUCTION

Despite decades of focused interventions, childhood undernutrition persists. Increasingly, nutrition campaigns in middle and lower income countries are advocating multidimensional approaches. One core strategy is to focus on first 1,000 days of life, as a phase influenced by quality of child feeding practices in ways critical to children's health and growth (Manikam et al., 2018; Burchi, Fanzo & Frison, 2011; World Bank 2008). Globally accepted infant and young child feeding guidelines recommend that neonates be breastfed immediately and continue exclusive breastfeeding until 6 months of age (World Health Organization [WHO], 2003). Starting at 6 months, mother's milk alone cannot meet the growing infant's caloric and micronutrient needs (WHO, 2001). Therefore, gradual introduction of optimally diversified foods is recommended at 6 months, alongside continued breastfeeding up to 2 years of age (Na, Aguayo, Arimond, & Stewart, 2017; Black et al., 2008; Begin et al., 1999). Adequate and diverse complementary feeding among young children is known to be the most important window to mitigate early childhood malnutrition (Na, Aguayo, Arimond, & Stewart, 2017).

Dietary diversity among infants and young children has been associated with maternal and child characteristics, household resources, and other sociocultural factors (Rakotonirainy et al., 2018; Kumera, Tsedal & Ayana, 2018; Santoso et al. 2019). Maternal education, awareness, and knowledge about child feeding practices are recognized factors affecting child's dietary diversity (Ickes et al., 2015). Among household resources, access to adequate and safe water is known to shape child nutrition and health outcomes, primarily through sanitation and hygiene pathways (Cumming & Cairncross, 2016; Subbaraman & Murthy 2015; Dewey & Mayers, 2011). However, a more direct role of water in child nutrition—specifically in infant and young child dietary diversity—remains largely unexplored. This is despite the fact that the literature linking water and food security does provide a sound basis for theorizing the possible role that household water might have in shaping child's access to dietary diversity.

Based on this literature, we can broadly identify three ways in which water might affect child's dietary diversity. First, regional water availability can affect child's dietary diversity, through its effect on household food security (Misra, 2014; United Nations Educational, Scientific, and Cultural Organization, 2012). This association has primarily been viewed from a community or productionist perspective, emphasizing production or availability of foods in the wake of growing demographic pressure and climate change (Sabo et al., 2017; Suweis, Rinaldo, Maritan, & D’Odorico, 2013; Qureshi, Hanjra, & Ward, 2013). Water availability affects household food diversity through food choice because in water‐scarce contexts, people may prefer to grow and eat less water‐intensive food (Mabhaudhi et al., 2016; High Level Panel of Experts [HLPE], 2015; Field, 1987). Water‐rich regions have opportunities for greater crop diversification and multicropping because they can more easily grow foods such as soybean, groundnut, and maize (Mabhaudhi et al., 2016). This increases the diversity of foods available in the food environment for purchase in the market and grown on‐plot within the household and reduces child malnutrition (Bezner‐Kerr, Berti, & Shumba 2011; HLPE, 2015). Greater local availability of water also increases the likelihood of households having water‐based animal foods such as fish in their diet (Hoekstra, 2012), thus increasing consumption diversity.

Second, at the household level, water access can affect dietary diversity through budget constraints that may force low‐resource households to choose between buying water, food, or fuel. When they purchase water, this leaves insufficient funds for acquiring an adequate and diverse diet (Cumming & Cairncross, 2016; Collins et al., 2019; Mason, 2015). Third, water at the household level may affect child's dietary diversity because water is very important in preparation of household foods (Wutich et al., 2017; Wutich & Brewis, 2014). Complementary foods have high water requirements (Wichmann & Voyi, 2006). Mothers have reported increased demand for water during the first 1,000 days of child life, for preparation of complementary foods and breastfeeding (Collins et al., 2019). An assessment of recipes for complementary foods prepared in low‐ and middle‐income countries identified water as a critical ingredient commonly comprising 70% to as high as 90% of ingredient proportions (Ferguson & Lehrfeld, 1998). Not having enough water to prepare complementary foods in this appropriate manner was the main reason cited for feeding infants less‐preferred foods in substitution in a recent cross‐cultural study of 19 sites in 16 low‐ and middle‐income countries (Schuster, Butler, Wutich, Miller, Young, & HWISE Consortium, n.d.). All these water–food and child feeding linkages are likely mediated by gender role arrangements given that women are usually managing both household water (Das & Safini, 2018) and child care (Rakotonirainy et al., 2018) duties in many parts of the world, including India.

Accordingly, we propose that inadequate availability and access to water is a key factor affecting child's consumption of minimum standard of dietary diversity—consuming foods from at least four of seven food groups as per WHO (2008). We test the three pathways outlined above that may link water insecurity to child's dietary diversity using large‐scale, nationally representative data for India. First, we test the proposition that low household water access lowers a child's achievement of minimum dietary diversity (MDD) standards. We also test that the relative effect of water access on households varies based on the general diversity of diets. Here, we engage primarily in theory building because we cannot precisely predict about the direction of this effect, for example, the suppressive effect of inadequate water access may be more pronounced for diets already higher or lower in diversity. Second, we test the proposition that low regional water availability is associated with lower dietary diversity. Notably, these proposed pathways are distinct from the WASH (water grouped with sanitation and hygiene) mechanism that focuses on effects of safe drinking water, sanitation, and hygiene practices on child nutrition (e.g., Troeger et al., 2018; Gautam et al., 2017; Wolf et al., 2014; Fink et al., 2011; Fewtrell et al., 2007; Torlesse et al., 2016).

### 1.1 Water and dietary diversity in India

India is home to the largest number of malnourished children in absolute terms. Nearly 38% of children under 5 years of age in India are stunted, or too short for their age, a commonly accepted indicator of childhood nutrition (Global Nutrition Report, 2018). Access of children to a minimally diverse diet is also very low in India; only 22% of children aged 6–23 months meet this metric (International Institute for Population Sciences [IIPS] & ICF, 2017; Menon et al., 2015). This figure masks significant class, rural–urban, and age‐based inequalities in accessing minimum dietary diversity (IIPS & ICF, 2017; Bentley et al., 2015).

Child's access to minimally diverse diet and nutrition is also variable across regions of India. In terms of child nutrition, southern states of India perform better than northern and central states (IIPS & ICF, 2017). Western states (except Goa) have mediocre achievement in this regard. Eastern states have very high level of child undernutrition (Pathak & Singh, 2011). Similar variation is reflected in child's access to dietary diversity (Raykar, Majumder, Laxminarayan, & Menon, 2015). Southern and western states have generally higher levels of dietary diversity compared with eastern and northern states (Borkotoky, Unisa, & Gupta, 2018; Das, 2014; IIPS & ICF, 2017).

Access to safe water is recognized as one of the key factors underlying high malnutrition in India through hygiene and sanitation mechanisms (Reddy et al., 2017; Flederjohann et al., 2015; Ravillion & Jalan, 2003; Million Death Study Collaborators, 2010; Rah et al., 2015; IIPS & ICF, 2017; Ngure et al., 2014). However, the role of water conceptualized more broadly in shaping children's dietary diversity remains unexplored. India is considered as a water stressed country (UN‐IDfA; Croninet al., 2016). A total of 54% of India faces high to extremely high stress in terms of surface water (Shiao et al., 2015). Most regions of India suffer from problems in terms of water quality, quantity, or both. The majority of northwest and Central India are water stressed, whereas South India—except the water abundant state of Kerala—has low to medium water availability (Piesse, 2017; Chakrapani, 2014; ADB, 2007).

This regional variation in water availability in India provides a suitable context for discerning the role that both household water access and regional availability might play in shaping children's dietary diversity. Here, we examine the effect of water availability and access on an important infant and young child feeding indicator—minimum dietary diversity (MDD)—in context of India using the nationally representative Demographic and Health Survey (DHS) data.

## 2 DATA AND METHODS

The data has been obtained from 2015–2016 India DHS, a nationally representative survey that provides comprehensive information on child care, child feeding practices, and relevant socio‐demographic information on the household. The sample covers children aged 6–23 months (*N* = 69,841), nested in relatively smaller number of households; each household may contain more than one child in the sampled age range.

### 2.1 Outcome variable: Minimum dietary diversity

Dietary diversity is in the pathway of child feeding and child nutrition—which is not limited to stunting and is inclusive of micronutrient deficiencies. Failure to consume MDD is associated with lower micronutrient intake (Kennedy, 2009). Therefore, we have chosen not to use child linear growth or stunting as an outcome in our analysis as we are focused on explicating the degree to which water affects what foods are available to and prepared in the household for infants and young children. This approach aligns with recent scholarship recognizing that a focus on linear growth and stunting as ultimate nutrition outcomes “discounts the importance of other outcomes” and risks overlooking the “positive, meaningful, and observable effects before linear growth improves” of other interventions (see Leroy & Frongillo, 2019).

Accordingly, our outcome variable is whether or not a child achieves minimum standards of dietary diversity as estimated from data collected through daily recall. This is a dichotomous variable taking the value “1” if child achieves MDD and “0” if child does not achieve MDD. According to WHO (2008), a child is classified as achieving MDD if during the previous day s/he was fed with at least four of seven food groups: grains, roots, and tubers; legumes and nuts; dairy products (milk, yogurt, and cheese); flesh foods (meat, fish, poultry, and liver/organ meats); eggs; vitamin A‐rich fruits and vegetable; and other fruits and vegetables.

### 2.2 Explanatory variables

#### 2.2.1 Household water access

Household water access is a key dimension of water insecurity (Jepson et al., 2017; Wutich et al., 2017) and includes dimensions of sufficient quantity, time to fetch water, and meeting household needs. We characterize water access levels based on an assessment of the household's water sources. The DHS solicits information on household's water sources through the question, “What is the main source of water used by your household for other purposes such as cooking and handwashing?” The response options include piped water, piped into dwelling, piped into yard/plot, bottled water, piped to neighbour, public tap, tube well water, tube well or borehole, protected well, protected spring, tanker truck, dug well, unprotected well, unprotected spring, river/dam, rainwater, and others. Following WHO standards described in Howard and Bartram (2003), water access based on levels of needs met is defined as (a) optimal—when all consumption and hygiene needs are met, (b) intermediate—when consumption needs are assured and basic personal and food hygiene are met, and (c) basic or no access—when consumption needs are difficult to fulfil and hygiene needs may or may not be assured.

Building on the WHO/UNICEF Joint Monitoring Program ladder for household water services (WHO/UNICEF 2017), we recoded the source of water variable—our main independent variable—in the DHS data in following manner:

1. Optimal access—where sources of water include piped water, piped into dwelling, piped into yard, and bottled water. We expect that among the available sources listed, these four sources are most likely to ensure water access in desirable quantity and quality, meeting all consumption and hygiene needs in comfortable manner.
2. Intermediate access—where sources of water include piped to neighbour, public tap, tube well water, tube well or borehole, protected well, protected spring, and tanker truck. These sources are more likely to assure that consumption needs are met and basic hygiene needs are likely to be met because even public sources are also protected.
3. Basic or no access—where sources of water include dug well, unprotected well, unprotected spring, river/dam, rainwater, and others. In these cases, we expect that consumption needs may be met with difficulty, whereas minimum hygiene norms can hardly be assured.

#### 2.2.2 Regional water availability

**Figure 1:**
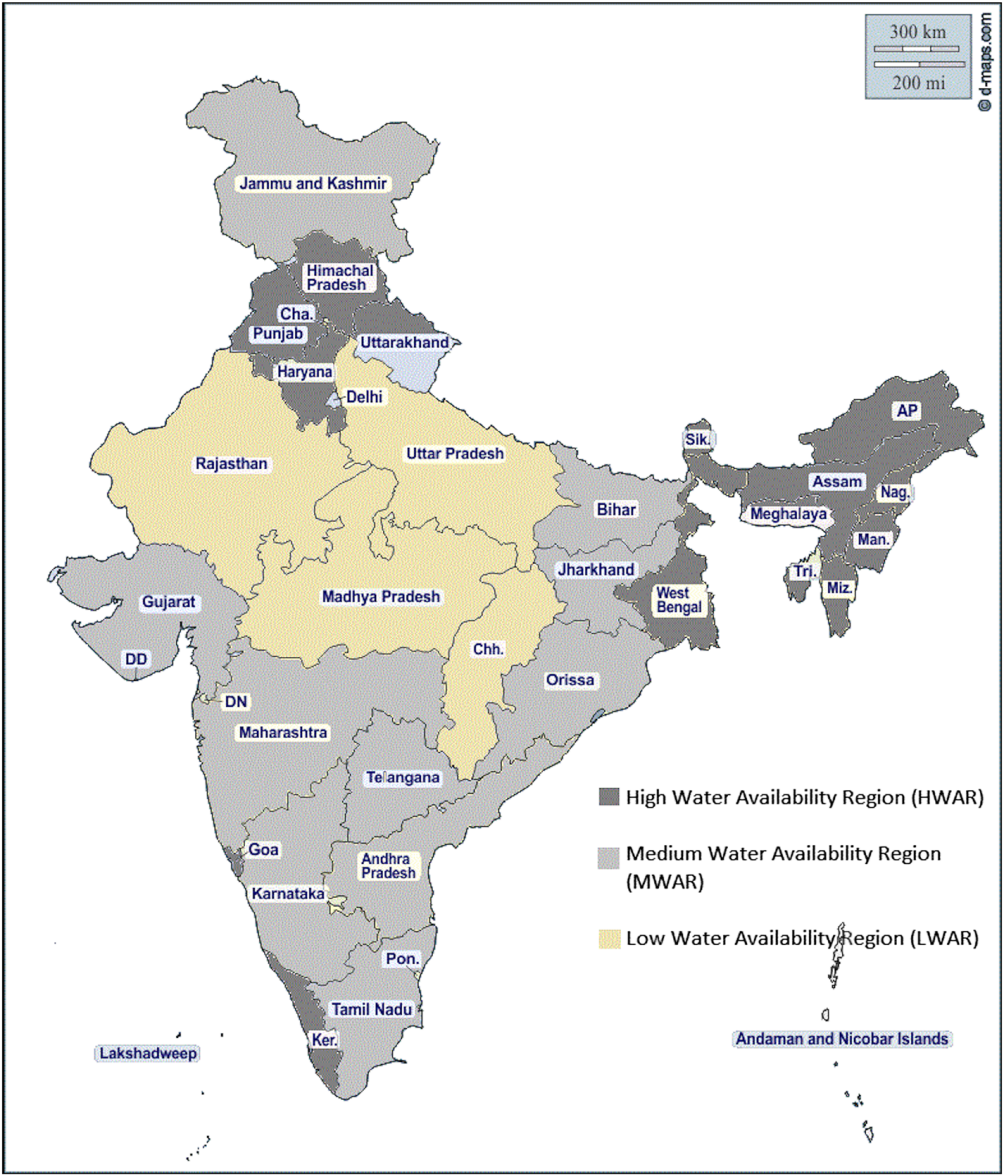
Regional classification of India based on the water availability index Source: Adapted from WBCSD (2019)

Our second water‐related predictor variable is relative regional water availability. For this, we classified India spatially on the basis of surface water availability index developed by WBCSD 1For methodology, see “India Water Tool” Technical Note: Available online at: http://www.indiawatertool.in/
 (2019). The value of the indicator ranges from −1 (low) to +1 (high) availability. The index classified India in six water availability regions, which have been recoded here in three categories (see map): low water availability region (LWAR), medium water availability region (MWAR), and high water availability region (HWAR). Households were then classified into these. Based on this scheme, HWAR included entire northeastern India (except Arunachal Pradesh), along with West Bengal, Himachal Pradesh, Punjab, Haryana, Uttarakhand, Kerala, and Goa (Figure 1). MWAR includes Bihar, Jharkhand, Odisha, Andhra Pradesh, Tamil Nadu, Maharashtra, Karnataka, Jammu and Kashmir, and Gujarat come under of India. Finally, Rajasthan, LWAR includes Telangana, Arunachal Pradesh, Madhya Pradesh, Chhattisgarh, and Uttar Pradesh.

#### 2.2.3 Other covariates

To take relative household material wealth into account in the models, we used categories based on the standard DHS‐constructed household wealth index. The wealth index is derived from housing characteristics and the number and kinds of consumer goods and amenities owned (Rutstein & Johnson, 2004). Household wealth categories are constructed by dividing the *n*‐ranked distribution into quintiles based on the absolute scores for the entire country sample.

Educated mothers are expected to be better aware on optimal child feeding practices. Our variable on maternal education is whether mother has completed primary, secondary, or high education, as given in DHS data. We also tested for the gender of head of household (female vs. male), given the relative social and economic vulnerability of the former compared with the latter.

Other variables included in the model are place of residence (rural or urban), child's age and gender, number of children under 5 years of age in the household, religion, and social group. In India, religious affiliation significantly shapes cultural preference for foods; for example, Hindus are likely to eat less animal product or eat it less frequently (Yadav, 2006; Bhalotra et al., 2009). We operationalized social group as household‐level caste or tribe (instead of the constitutional classification 2India is traditionally a caste stratified society. In support of affirmative action, the Indian constitution defines certain population groups as Scheduled Caste and Schedule Tribes, based on their social and economic status.) because dietary preferences are likely more variable between castes and tribes as compared with within castes (Ferro‐Luzzi, 1975).

Notably, we have not included variables on access to safe drinking water, hygiene, and sanitation because our outcome of interest is dietary diversity, upstream of measured nutrition status. This aligns with our objective to identify the role of household water access—not access to safe drinking water or associated hygiene practices (which we would expect to affect nutrition outcomes through the WASH pathways)—on child dietary diversity. Research on dietary diversity typically does not include sanitation or hygiene variables (see Rakotonirainy et al., 2018).

#### 2.2.4 Estimation method

Because our outcome variable is a dichotomous/binary variable, we use the probit regression to estimate the model. We also ran ordinary least square regression using child's dietary score as continuous dependent variable. However, the results—not reported here—did not differ from probit regression. As per the probit model, the probability that a child achieves minimum dietary diversity can be expressed as

(1) $$\text{Prob}\;\left(Y=1\right)=\alpha +\mathrm{\gamma X}+\varepsilon ,$$

where Y = 1, if the child was fed with minimum dietary diversity; Y = 0, otherwise; X is the vector of variables that could determine the probability that a child achieves MDD; α is the constant terms in the equations; γ is the vector of coefficients of explanatory variables in the equations; and ε is the error term.

We first estimate this model for all of India including various water availability regions as dummy variable. Then we also estimate separate models for each water availability region. The rationale is that in varying context of water availability, the relationship between household water access and child's access to MDD may vary.

Because our analysis covers the entire India wherein a case represents each child aged 6–23 months, we do not weight our analysis. Sample weights are used to account for survey design used for sample selection. However, as instructed in the technical guide to DHS, we do not need to use sample weights while estimating relationships such as performing regression models (Rutstein and Rojas, 2006). The data analysis has been conducted using Stata v16 software.

## 3 RESULTS

### 3.1 Sample characteristics

Table 1 shows the background characteristics of our sample of 69,841 children aged 6–23 months. Average child age is 14.4 months, and 48% of children are female. Approximately 27% of mothers have no formal schooling, 14% have completed primary school, 47% of mothers have completed secondary school, and 12% have attained higher education. The majority of our sample are Hindu (79%), from a caste (88%), and reside in a rural area (72%). The majority of households have intermediate access to water (62.8%); only 26.5% have optimal access to water. For regional distribution, 42% of children come from households in LWAR, 33% from MWAR, and 25% from HWAR.

**Table 1:** Socio‐demographic characteristics of sample of infant and young children aged 6–23 months

Variable	Proportion in percentage (*N* = 69,841)
Child achieved minimum dietary diversity	19.78
Maternal education	
Did not complete primary education	27.39
Primary educated	13.54
Secondary educated	47.38
High educated	11.69
Household religion	
Hindu	79.0
Muslim	16.5
Christian	2.0
Other	2. 5
Household social group	
Caste	88.1
Tribe	7.24
Other (neither caste nor tribe)	4.6
Household wealth class	
Lowest wealth class	24.4
Lower wealth class	21.7
Middle wealth class	20.4
Higher wealth class	18.5
Highest wealth class	15.0
Household water access	
Optimal	26.53
Intermediate	62.80
Basic or no access	10.65
Other household level covariates	
Female headed household	12.27
Reside in rural area	71.93
Child is female	47.67
Regional water availability	
High water availability region (HWAR)	41.96
Medium water availability region (MWAR)	33.30
Low water availability region (LWAR)	24.74

Only 20% of infants and young children in our sample achieved MDD standards (Table 1). The proportion of young children having achieved MDD is highest (23%) among households with optimal access to water, followed by those in household with intermediate water access (19.2%), and those in households with basic or no access to water (18%; Table 2). In terms of water regions, the proportion of children achieving MDD is the highest for HWAR followed by MWAR and LWAR. Chi‐squared measure of association between the percentage of children having achieved MDD and levels of household water access and water availability regions is significant at *p* < .01 (Table 2).

**Table 2:** Proportion (in %) of infant and young children aged 6–23 months achieving minimum dietary diversity by household water access and regional water availability categories (*N* = 69,841)

	Proportion who achieved minimum dietary diversity
Household water access	
Optimal	23.0 (0.0031)
Intermediate	19.22 (0.0018)
Basic or no access	18.0 (0.0048)
Pearson's χ^2^ (2)	276.09**
Regional water availability	
Low water availability region (LWAR)	13.7 (0.0038)
Medium water availability region (MWAR)	23.15 (0.0044)
High water availability region (HWAR)	27.9 (0.0073)
Overall	19.78 (0.0014)
Pearson's χ^2^ (2)	9481.7**

a *Note*. Standard deviation in parentheses.

* significant at.05 level.

** significant at.01 level.

**Figure 2:**
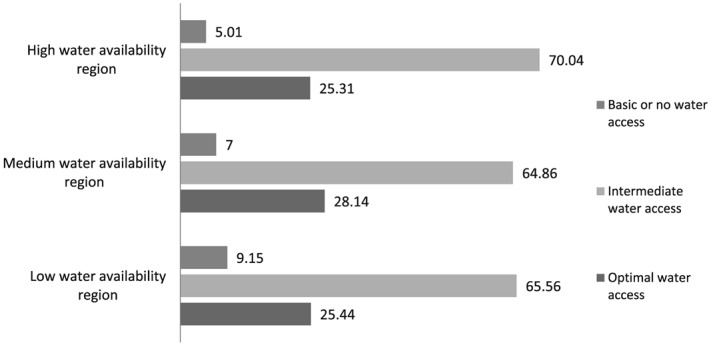
Proportion of households with basic, intermediate, and optimal household water access across low, medium, and high water availability regions in India

Figure 2 shows levels of household water access by each of the three water availability regions of India. HWAR region does not necessarily have highest proportion of households with optimal water access. In fact, proportion of households with optimal water access in LWAR and HWAR is nearly same at around 25%.

### 3.2 Results from probit regression

#### 3.2.1 Both household water access and regional water availability context are associated with child's access to dietary diversity

Table 3 gives results from probit regression on child's access to minimum dietary diversity. To account for heteroskedasticity in data, robust standard errors have been reported for all probit estimates. We tested overall model fit using Hosmer–Lemeshow test. With a significance value of *p* < .05, the test described our model as adequately describing the data.

**Table 3:** Estimates from probit regression predicting achievement of minimum dietary diversity standards for a child aged 6–23 months across India

*N* = 68,490	Marginal effect at means	Standard Error
Access to water (Ref: Optimal)		
Intermediate	−.0198**	.0037
Basic or no access	−.0209**	.0058
Maternal education (Ref: No formal schooling)		
Primary education	.0035	.0047
Secondary education	.0343**	.0039
High education	.0516**	.0063
Household's wealth class (Ref: Lowest wealth)		
Lower wealth class	.0096*	.0043
Middle wealth class	.0217**	.0047
Higher wealth class	.0301**	.0054
Highest wealth class	.0363**	.0064
Social group (Ref: caste)		
Tribe	.0234**	.0052
Neither caste nor tribe (other)	.0815**	.0081
Religion (Ref: Hindu)		
Muslim	.0479**	.0044
Christian	.0552**	.0067
Other	.0224**	.0076
Child's age	.0145**	.0002
Currently breastfeeding (Ref: No)		
Yes	−.0613**	.0043
Child's gender (Ref: Female)		
Male	.0025	.0028
Number of under five children in the household	−.0110*	.0065
Gender of household head (Ref: Male)		
Female	−.0264*	.0172
Type of residence (Ref: Rural)		
Urban	.0122*	.0038
Regional water availability (Ref: Medium water availability)		
Low water availability	−.0924**	.0047
High water availability	.0287**	.0061
Region*Rural/Urban (Ref: Medium water availability rural		
Medium water availability—urban	.0198**	.0186
Low water availability—rural	−.0890**	.0056
Low water availability—urban	−.1015**	.0083
High water availability—rural	.0394**	.0064
High water availability—urban	.0508**	.0113
	Wald χ^2^ (24) = 5706.75** Pseudo *R* ^2^ = .0873	

* significant at.05 level.

** significant at.01 level.

Household access to water appears as a significant predictor at *p* < .01 level of significance, though the size of effect is not big. As compared with children in households with optimal water access, a child in a household with intermediate water access and with basic or no water access has lower probability of achieving MDD by 1.9 and 2.1 percentage points, respectively. Regional water availability in India is statistically significant as well. As compared with a child in MWAR, a child in LWAR has 9.2 percentage point lower probability, and a child in HWAR has 3 percentage point higher probability of achieving MDD standards. We also introduced an interaction variable representing rural and urban areas of various water availability regions. As compared with a child in rural MWAR, a child in urban MWAR has 1.9 percentage point higher probability of achieving MDD. Similarly, a child in both rural and urban LWAR has 9–10 percentage point lower probability of receiving MDD when compared with a child in rural MWAR. Children from rural and urban HWAR have a 3.9 percent and 5.0 percent higher probability of achieving minimum dietary diversity standards as compared with rural MWAR.

**Figure 3:**
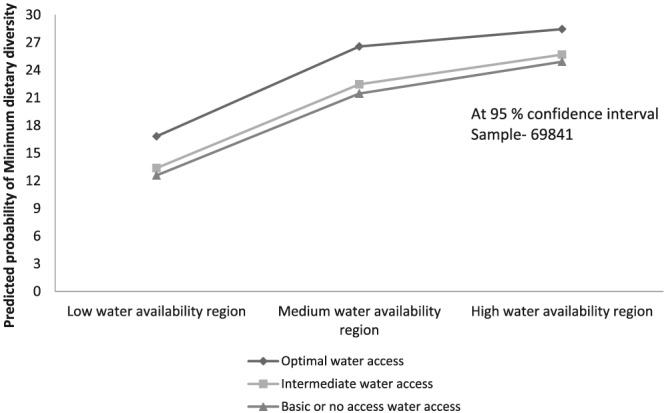
Predicted probability of a child aged 6–23 months achieving World Health Organization's minimum dietary diversity standards by household water access and regional water availability

Figure 3 shows predicted probability that a child achieves MDD by household water access level across the three water availability regions of India. Predicted probability of achieving the standards is the highest in case of households with optimal water access within HWAR (28.5%) followed by households with optimal water access in MWAR (26.5%; Figure 3). Households with basic water access in LWAR have the lowest probability of achieving MDD. Further, even within households with optimal water access in LWAR, the probability of child achieving MDD is lower than households with intermediate and limited water access in both HWAR and MWAR.

In terms of demographic factors related to gender, child in male headed households had a 2.6 percentage point higher probability of achieving MDD compared with child in female headed households. As compared with a child whose mother did not attend formal schooling, those with a secondary educated mother had 3.4 percentage point higher probability and those with high educated mother has 5.1 percentage point higher probability of achieving MDD standards.

A currently breastfed child had 6 percentage point lower probability of achieving dietary diversity standards. For each 1‐month increase in child age, their probability of achieving minimum standards increased by 1.4 percentage point. Number of children under 5 years of age in the household was also a significant predictor of a child achieving minimum dietary diversity, but child's gender is not.

Other household factors predicting a child's probability of achieving minimum dietary diversity included household wealth; a child from the middle, higher, and highest wealth class has 2.1, 3, and 3.6 percentage point higher probability, respectively, of achieving minimum standards compared with the lowest wealth class. Children in rural households has 1.2 percentage point lower probability compared with those in urban households. In terms of religion, as compared with a Hindu child, a child from any other religion has higher probability of achieving MDD. Similarly, child belonging to a caste group had lower probability of achieving MDD as compared with a child from a tribe or other group by 2.3 and 8 percentage points, respectively.

#### 3.2.2 The relationship between household water access and child's access to MDD is variable across regional water availability contexts

Table 4 shows results from separate probit model by regional water availability. In each water region, there is a significant association between household water access and child's probability of achieving MDD. Table 4 also gives test results on overall significance of household water access, and it appears to be significant at 0.01 level in each region. In LWAR and MWAR, the probability of child achieving MDD was lower in households with intermediate water access by 1.8 percentage point and 6.2 percentage point, respectively, compared with children in households with optimum water access. However, in HWAR, a child in household with intermediate water access had a 7.7 percentage point higher probability of achieving MDD as compared with those with optimal water access. In all water availability regions, there is however no significant difference in the probability of a child achieving MDD between households with optimal water access and household with basic water access. Other covariates appear to follow a pattern similar to the overall probit model including all regions (Table 3).

**Table 4:** Estimates from probit regression predicting achievement of minimum dietary diversity for a child aged 6–23 months across India across each of three regional water availability contexts

	Low water availability region *N* = 28,165	Medium water availability region *N* = 22,880	High water availability region *N* = 17,445
	Marginal effect at means (Standard error)	Marginal effect at means (Standard error)	Marginal effect at means (Standard error)
Source of water (Ref: Optimal)			
Intermediate	−.0183** (.0053)	−.0626** (.0081)	.0775** (.0079)
Basic or no access	−.0017 (.0086)	−.0146 (.0120)	−.0057 (.0095)
Maternal education (Ref: No education)			
Primary education	.0121* (.0058)	−.0249 (.0114)	.0239* (.0104)
Secondary education	.0370** (.0049)	.0433** (.0089)	.0401** (.0083)
High education	.0346** (.0083)	.0980** (.0151)	.0704 ** (.0132)
Household's wealth (Ref: Lowest wealth)			
Lower wealth class	.0098 (.0056)	.0410* (.0099)	−.0057 (.0095)
Middle wealth class	.0082 (.0061)	.0745** (.0105)	.0069 (.0106)
Higher wealth class	.0249** (.0071)	.0914** (.0125)	.0128(.0117)
Highest wealth class	.0212** (.0084)	.1024** (.0159)	.0110 (.0133)
Social group (Ref: Caste)			
Tribe	.0576** (.0071)	−.0303** (.0108)	.0344 (.0230)
Other	.0654** (.0192)	.0442** (.0141)	.0911** (.0176)
Religion (Ref: Hindu)			
Muslim	.0161** (.0061)	.0869** (.0101)	.0183* (.0090)
Christian	.1271** (.0170)	−.0017 (.0189)	.0815** (.0333)
Other	.1097** (.0153)	.0316** (.0227)	−.0325** (.01190)
Child's age	.0105** (.0003)	.0171** (.0006)	.0145** (.0005)
Currently breastfeeding (Ref: No)			
Yes	−.0406** (.0058)	−.1249** (.0099)	−.0542** (.0096)
Child's gender (Ref: Female)			
Male	−.0053 (.0038)	.0024 (.0065)	−.0038 (.0062)
Number of under five children in the household			
	.0014 (.0021)	.0026 (.0040)	−.0005 (.0034)
Gender of household head (Ref: Male			
Female	.0010 (.0067)	.0073 (.0109)	−.0079 (.0080)
Type of residence (Ref: Urban)			
Rural	−.0126** (.0055)	−.0110* (.0083)	−.0342** (.0088)
Overall significance of household water access	χ^2^ (2) = 15.87**	χ^2^ (2) = 9.71**	χ^2^ (2) = 30.25**
	Wald χ^2^ (20) = 1458.11** Pseudo *R* ^2^ = .066	Wald χ^2^ (20) = 1986.71** Pseudo *R* ^2^ = .089	Wald χ^2^ (20) = 1289.46** Pseudo *R* ^2^ = .055

* significant at.05 level.

** significant at.01 level.

## 4 DISCUSSION

Our analysis contributes to existing literature by clearly establishing the basic association between water and child's likelihood of achieving MDD, which may shape child nutrition outcomes. Our finding that suboptimal household water access is associated with lower probability of a child achieving MDD advances the emergent body of research that offers insights into a few ways through which the linkage between water access and dietary diversity unfold (e.g., Collins et al., 2019; Gibson, Ferguson, & Lehrfeld, 1998; Schuster, Butler, Wutich, Miller, Young, & HWISE Consortium, n.d). Household water access can affect child's dietary diversity also through gendered and competing demands on caregiver time. A 19‐site cross‐cultural study identified household water insecurity as qualitatively associated with delays in infant feeding, as the caregiver spent more time obtaining and managing water and less time preparing food for feeding the infant (Schuster, Butler, Wutich, Miller, Young, & HWISE Consortium, n.d.). Our finding that child in female headed household has lower probability of achieving minimum dietary diversity could also be because women are more time constrained due to multiple responsibilities. This is more applicable in Indian context where a female headed household usually represents a household where the female head is a widow or the male head of household has otherwise left so that her responsibilities are not shared by a partner.

Our second finding that at regional level, water availability can affect child's dietary diversity by altering the food availability dimension through its contribution to agricultural and allied activities, is also in conformity with the water–food literature (Mabhaudhi et al., 2016; HLPE, 2015). Our analysis further adds to the literature by suggesting how the relationship between household water access and a child achieving MDD varies regionally. Particularly, living in a higher water region suppresses or buffers the ill‐effect of low household water access on children's dietary diversity. This could be a possible explanation for our finding that the child in a household located in HWAR but having intermediate water access has higher probability of achieving minimum dietary diversity standards compared with the child in a household with optimal water access, located in LWAR. More than half of HWAR is covered by the northeastern states of India, which is a water‐rich region with heavy rainfall. However, this region is primarily hilly and thus relies more on tube wells and natural water sources (e.g., water bodies and springs). Hence, despite being in HWAR, as per our measure of household water access, the majority (70%) of households in northeastern India have intermediate access to water. Alongside this, the proportion (~40%) of children achieving minimum dietary diversity in this region is also higher than Indian average (IIPS & ICF, 2017). Being in HWAR possibly compensates for restrictions posed due to suboptimal water access.

Our finding that breastfeeding children are less likely to achieve MDD fits with the premise that as complementary foods are included in child's diet, breastfeeding transitions to a more supportive role until complete weaning (Belew et al., 2017). Recent qualitative work has identified that in some contexts, women elect to breastfeed as a replacement for complementary foods when there is insufficient water to prepare these nonbreastmilk foods (Schuster, Butler, Wutich, Miller, Young, & HWISE Consortium, n.d.). At age 6 months and beyond, this may be cause for concern because breastmilk alone cannot meet the child's micronutrient and macronutrient needs; children aged 6–11 months experience significant challenges in accessing sufficient micronutrients in the transition to nonbreastmilk foods (Dewey, 2013). This suggests an aligned hypothesis for further testing that mothers may elect to breastfeed longer or more frequently when they are unable to prepare minimally required complementary foods because household has inadequate water access to prepare foods or because of time/other constraints posed by inadequate household water access.

Finally, by identifying the role of water in shaping children's access to dietary diversity, our analysis provides an additional window for nutrition policy and intervention. Optimal access to water can improve child's likelihood of achieving minimum standards of dietary diversity and therefore, can have potential effect on child nutrition through pathways separate from WASH. Further research is warranted to explore how household water access and community/regional water context interact and are mediated by the gender dimension to shape child's dietary outcomes and in turn child nutrition.

### 4.1 Limitations

The cross‐sectional nature of the data constraints intertemporal prediction of proposed relationships. We recognize that even though our results show statistically significant association between household water access and the probability of child achieving MDD, the magnitude of this difference by water access is low. However, our findings unfold possible pathways linking water to MDD, which can be further examined through mixed method research.

Our measure of household water access—based on classification of water sources—overlooks other challenges to water access such as quality and time to access water that may vary within and between water sources. For example, a study from Uganda (Lauer et al., 2018) showed that water from improved sources were contaminated (Lauer et al. 2018). However, the measure of water access used in this study is considered by water experts as an acceptable proxy in water insecurity studies (Wutich et al., 2017).

The data set did not provide a measure of caregiver's time allocation across household water management or child care activities, which constrains our insight into the underlying relationships. Furthermore, we have used child's achievement of MDD as a binary variable that might have compromised some precision in data. Finally, we do not have data to show how water access affected cooking patterns and irrigation activities.

## 5 CONCLUSIONS

Our analysis provides evidence that inadequate water access—associated as it may be with low household wealth—provides additional pathway to child nutrition as defined in terms of access to minimum dietary diversity. Suboptimal water access may intersect with limited household resources to reinforce reduced dietary diversity, especially in low overall water availability regions, and for household where resource shortages are more concentrated. To the extent child's dietary diversity is associated with sufficient macronutrient and micronutrient consumption, this may have implications for child nutritional status. This latter part may be an area for further research. Our findings also indicate the need for more elaborate data collection on gendered roles and water management.

## CONFLICT OF INTEREST

The authors declare that they have no conflicts of interest.

## AUTHOR CONTRIBUTIONS

ABRS and NC conceived the study; NC designed the analysis and analysed the data; and NC, RS, AB, and AW prepared and approved the draft.
